# Metatranscriptomics Reveals Sequential Expression of Genes Involved in the Production of Melanogenesis Inhibitors by the Defined Microbial Species in Fermented Unpolished Black Rice

**DOI:** 10.1128/spectrum.03139-22

**Published:** 2023-03-02

**Authors:** Orrarat Sangkaew, Pinidphon Prombutara, Sittiruk Roytrakul, Chulee Yompakdee

**Affiliations:** a Department of Microbiology, Faculty of Science, Chulalongkorn University, Pathumwan, Bangkok, Thailand; b Omics Science & Bioinformatics Center, Faculty of Science, Chulalongkorn University, Pathumwan, Bangkok, Thailand; c Functional Ingredients and Food Innovation Research Group, National Center for Genetic Engineering and Biotechnology, National Science and Technology Development Agency, Klong Luang, Pathumthani, Thailand; University of Minnesota Twin Cities

**Keywords:** metatranscriptomics, melanogenesis inhibition, fermentation, starter, fermented black rice

## Abstract

Fermented products require metabolic enzymes from the microbial community for desired final products. Using a metatranscriptomic approach, the role of microorganisms in fermented products on producing compounds with a melanogenesis inhibition activity has not yet been reported. Previously, unpolished black rice (UBR) fermented with the E11 starter containing Saccharomyces cerevisiae, Saccharomycopsis fibuligera*, Rhizopus oryzae*, and Pediococcus pentosaceus (FUBR) showed potent melanogenesis inhibition activity. This study aimed to investigate the function of these defined microbial species in producing melanogenesis inhibitors in the FUBR using a metatranscriptomic approach. The melanogenesis inhibition activity increased in a fermentation time-dependent manner. Genes related to melanogenesis inhibitors synthesis such as carbohydrate metabolism, amino acids synthesis, fatty acids/unsaturated fatty acids synthesis, and carbohydrate transporters were analyzed. Most genes from *R. oryzae* and P. pentosaceus were upregulated in the early stage of the fermentation process, while those of S. cerevisiae and *S. fibuligera* were upregulated in the late stage. FUBR production using different combinations of the four microbial species shows that all species were required to produce the highest activity. The FUBR containing at least *R. oryzae* and/or P. pentosaceus exhibited a certain level of activity. These findings were in agreement with the metatranscriptomic results. Overall, the results suggested that all four species sequentially and/or coordinately synthesized metabolites during the fermentation that led to a FUBR with maximum melanogenesis inhibition activity. This study not only sheds light on crucial functions of certain microbial community on producing the melanogenesis inhibitors, but also paves the way to initiate quality improvement of melanogenesis inhibition activity in the FUBR.

**IMPORTANCE** Fermentation of food is a metabolic process through the action of enzymes from certain microorganisms. Although roles of the microbial community in the fermented food were investigated using metatranscriptomic approach in terms of flavors, but no study has been reported so far on the function of the microorganisms on producing compounds with a melanogenesis inhibition activity. Therefore, this study explained the roles of the defined microorganisms from the selected starter in the fermented unpolished black rice (FUBR) that can produce melanogenesis inhibitor(s) using metatranscriptomic analysis. Genes from different species were upregulated at different fermentation time. All four microbial species in the FUBR sequentially and/or coordinately synthesized metabolites during fermentation that led to a FUBR with maximal melanogenesis inhibition activity. This finding contributes to a deeper understanding of the roles of certain microbial community during fermentation and led to the knowledge-based improvement for the fermented rice with potent melanogenesis inhibition activity.

## INTRODUCTION

Fermentation is a metabolic process that produces chemical changes in organic substrates through the action of enzymes. Many fermentation processes involve a diverse range of microorganisms, including lactic acid bacteria, yeasts, and/or molds (1, 2). The core microorganisms play critical roles in determining the characteristics of the flavor metabolites and nutritional value of the obtained fermentation products. This process is widely used to improve the shelf-life, safety, nutritional and functional properties, and organoleptic properties of food products (1). Many reports have demonstrated that fermentation not only enhances the biological activity of the raw material but can also lead to new biological activities (3, 4). Hence, gaining insight into the functional role of microbial communities and the mechanism of metabolite formation involved in fermentation has become interesting.

Due to the development of the “omics” technologies, metatranscriptomics constitutes an ideal tool for studying microbial ecology, as it directly analyzes mRNA from environments and provides information not only on the microbial community composition but also on the active members and their specifically expressed enzymes (5). This technology has been widely used to study gene expression (6). Recently, several studies have also utilized metatranscriptomics to identify microbial communities and their functions in fermented foods. Zhang et al. (2021) used a metatranscriptomic approach to comprehensively explore the functional and enzyme dynamics of microbes during noni fruit fermentation. Moreover, the active microbial community and their metabolic function in the fermentation of soybean paste, cocoa, Chinese liquor, and Chinese Sichuan Paocai were studied using a metatranscriptomic approach (7–10). These studies have provided a better understanding of the microbial community structure and their metabolic function in fermented foods. Many works regarding fermented foods have focused on their flavor formation; however, there is no study using a metatranscriptomic approach to focus on melanogenesis inhibition activity.

Fermented rice products have been considered as dietary agents to promote the growth development of malnutritioned children, activate bacterial activity, and their use as a dietary supplement. Moreover, fermented rice products have been reported to have enhanced antioxidant, anti-cancer, anti-inflammatory bowel diseases, anti-diabetes, and anti-aging activities (11).

Melanogenesis is the complex process for melanin pigments synthesis, that is the cause of skin pigmentation. Among enzymes in this process, tyrosinase is the key enzyme that catalyzes the hydroxylation of tyrosine to 3,4-dihydroxyphenylalanine (DOPA) as the rate-limiting step and the subsequent oxidation of DOPA to dopaquinone (12). Melanin has important role in the skin protection from UV ray; however, the accumulation of melanin leads to esthetic problem such as melasma and freckle (13). Therefore, melanogenesis inhibitors are necessary for hyperpigmentary skin disorders treatment. Many studies investigated the efficacy of fermented products on melanogenesis and tyrosinase inhibition such as fermented product of soybean, *Viola mandshurica*, *Rhodiola rosea*, and *Lonicera japonica* (14–16). In a previous study, the liquid from unpolished black rice (UBR) fermented with the selected microbial starter (*loogpang*) E11 for 12 d (FUBR) showed a melanogenesis inhibitory activity (17). In addition, the liquid obtained from the FUBR using a mixture of the defined microbes isolated from the selected *loogpang* E11 starter (containing S. cerevisiae, *S. fibuligera*, *R. oryzae*, and P. pentosaceus) for 12 days showed a similar anti-melanogenesis activity as that from the original starter (17). Therefore, to comprehensively understand the roles of these four types of microbes in the E11 starter in terms of contributing to melanogenesis inhibitor production in the FUBR, a metatranscriptomic approach was used. In this study, the total RNA of the microbiome at different fermentation times was extracted, and a metatranscriptomic approach was applied to globally and comprehensively analyze the microbial community structure and metabolic functions during fermentation of UBR. The metabolic pathways and enzyme activities of the core microorganisms during the UBR fermentation were systematically analyzed, which will provide a theoretical basis and technical support for product quality improvement and new product development.

## RESULTS

### Melanogenesis inhibition activity of the FUBR from the E11 starter at different fermentation times.

**FIG 1:**
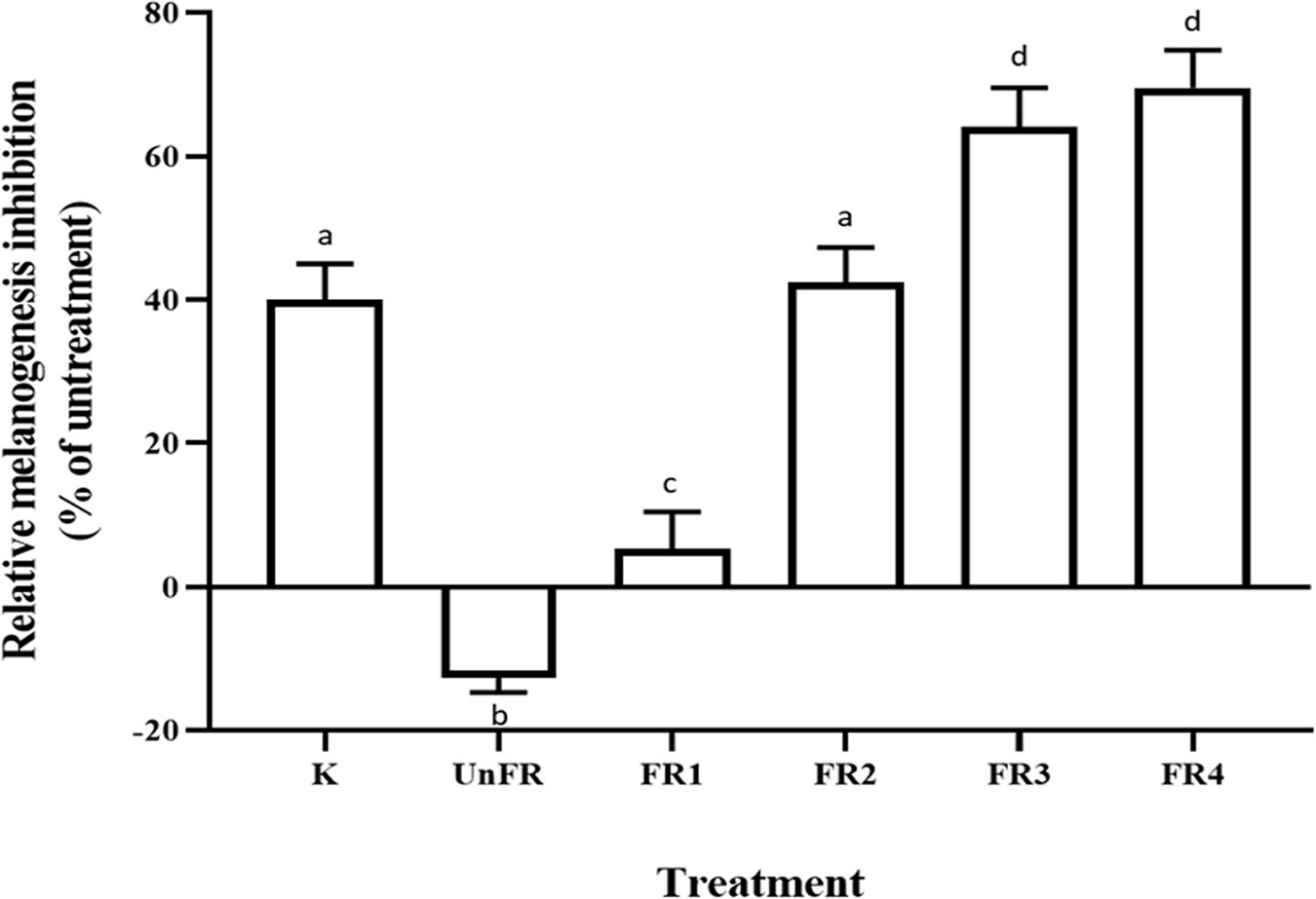
Inhibitory effect of FUBR on melanogenesis in B16F10 melanoma cells. B16F10 melanoma cells were treated with 1 mM kojic acid (K) as a positive control and FR-Liq from different fermentation times: 0, 3, 6, 9, and 12 days designated as UnFR, FR1, FR2, FR3, and FR4, respectively. The results are expressed as a percentage of the untreated group, and the data are the mean ± SEM from three independent experiments. Bars marked with different letters are significantly different (*P* < 0.05).

To determine the melanogenesis inhibition activity, FUBR samples obtained from E11 starter at different fermentation times (UnFR, FR1, FR2, FR3, and FR4; which corresponded to unfermented sample, and fermented liquid from day 3, 6, 9, and 12, respectively) was evaluated with the B16F10 melanoma cell. The results indicated that all the FR samples significantly inhibited melanin production in B16F10 melanoma cells in a fermentation-time-dependent manner. In contrast to the FR samples, the UnFR sample showed no melanogenesis inhibition activity against the B16F10 cells (Fig. 1).

### Functional profiling of FUBR.

**FIG 2:**
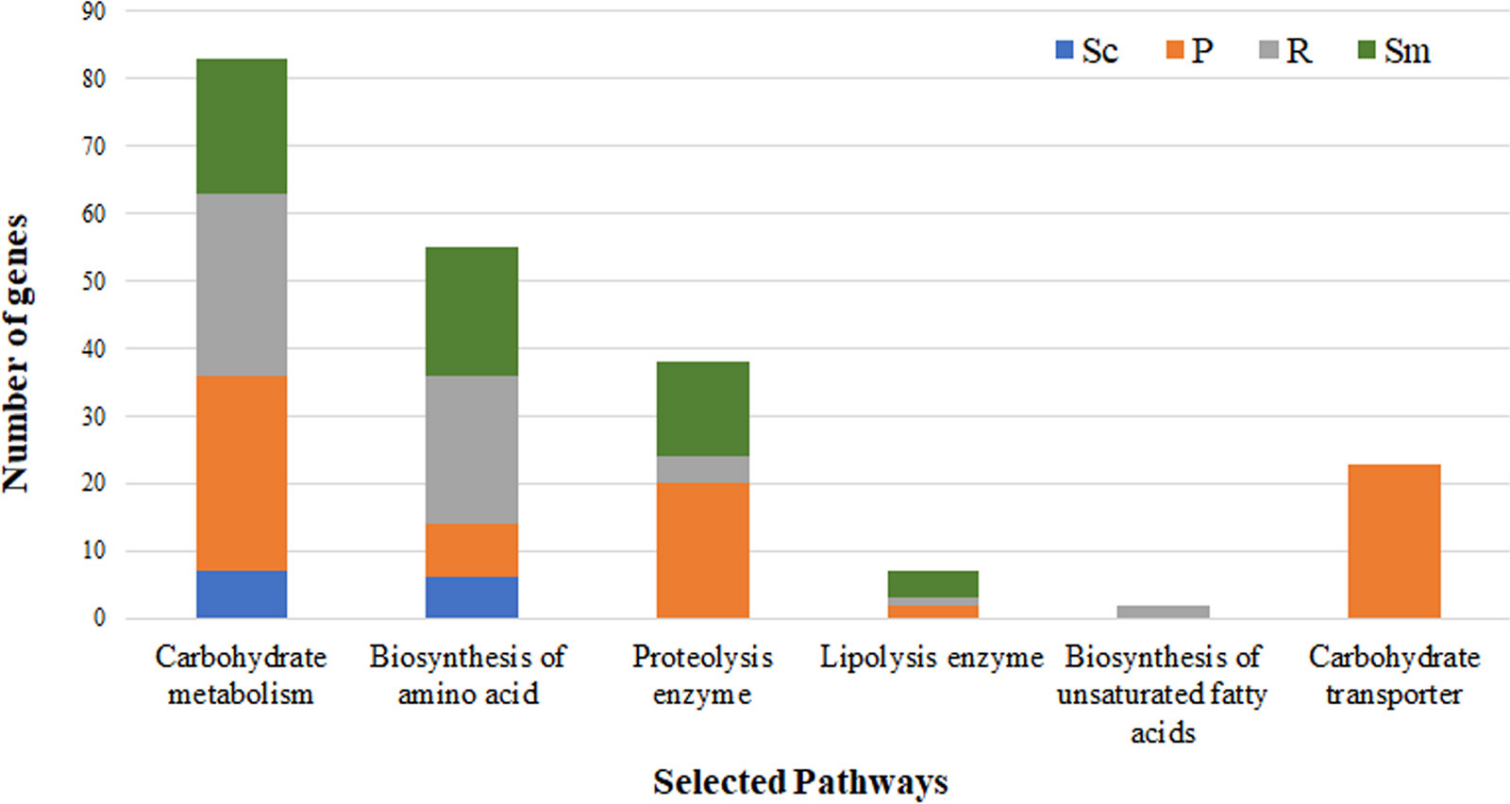
Classification of the transcripts encoding for the identified enzymes in the FUBR into selected pathways. The enzymes from each microorganism were clustered corresponding to selected pathways (carbohydrate metabolism, biosynthesis of amino acids, proteolysis enzymes, lipolysis enzymes, biosynthesis of unsaturated fatty acid and carbohydrate transporters). Sc, Sm, R, and P represent S. cerevisiae, *S. fibuligera*, *R. oryzae*, and P. pentosaceus, respectively.

To determine the active genes in the UBR fermentation process, the FUBR at different fermentation times (UnFR, FR1, FR2, FR3, and FR4) were analyzed using a metatranscriptomic approach. Based on the KEGG database, this study focused on genes related to carbohydrate metabolism, biosynthesis of amino acids, proteolysis enzymes, lipolysis enzymes, biosynthesis of unsaturated fatty acids, and carbohydrate transporters. The profiles of genes related to these selected pathways are shown in Fig. 2. Among these six selected pathways, carbohydrate metabolism had the most abundant genes, followed by genes related to the biosynthesis of amino acids, proteolysis enzymes, carbohydrate transport, lipolysis enzyme, and biosynthesis of unsaturated fatty acids, respectively. Additionally, the metatranscriptomic results showed that the number of genes related to carbohydrate metabolism were mostly obtained from P. pentosaceus and *R. oryzae*, whereas the largest number of genes related to the biosynthesis of amino acids and unsaturated fatty acids came from *R. oryzae*. *R. oryzae* was the only source of genes related to unsaturated fatty acids biosynthesis. The largest number of genes related to proteolysis enzymes and carbohydrate transporters were obtained from P. pentosaceus. P. pentosaceus was the only species found to contribute to carbohydrate transporters. The number of genes related to lipolysis enzymes were mostly obtained from *S. fibuligera* (Fig. 2).

### Identification of genes related to carbohydrate metabolism.

**FIG 3:**
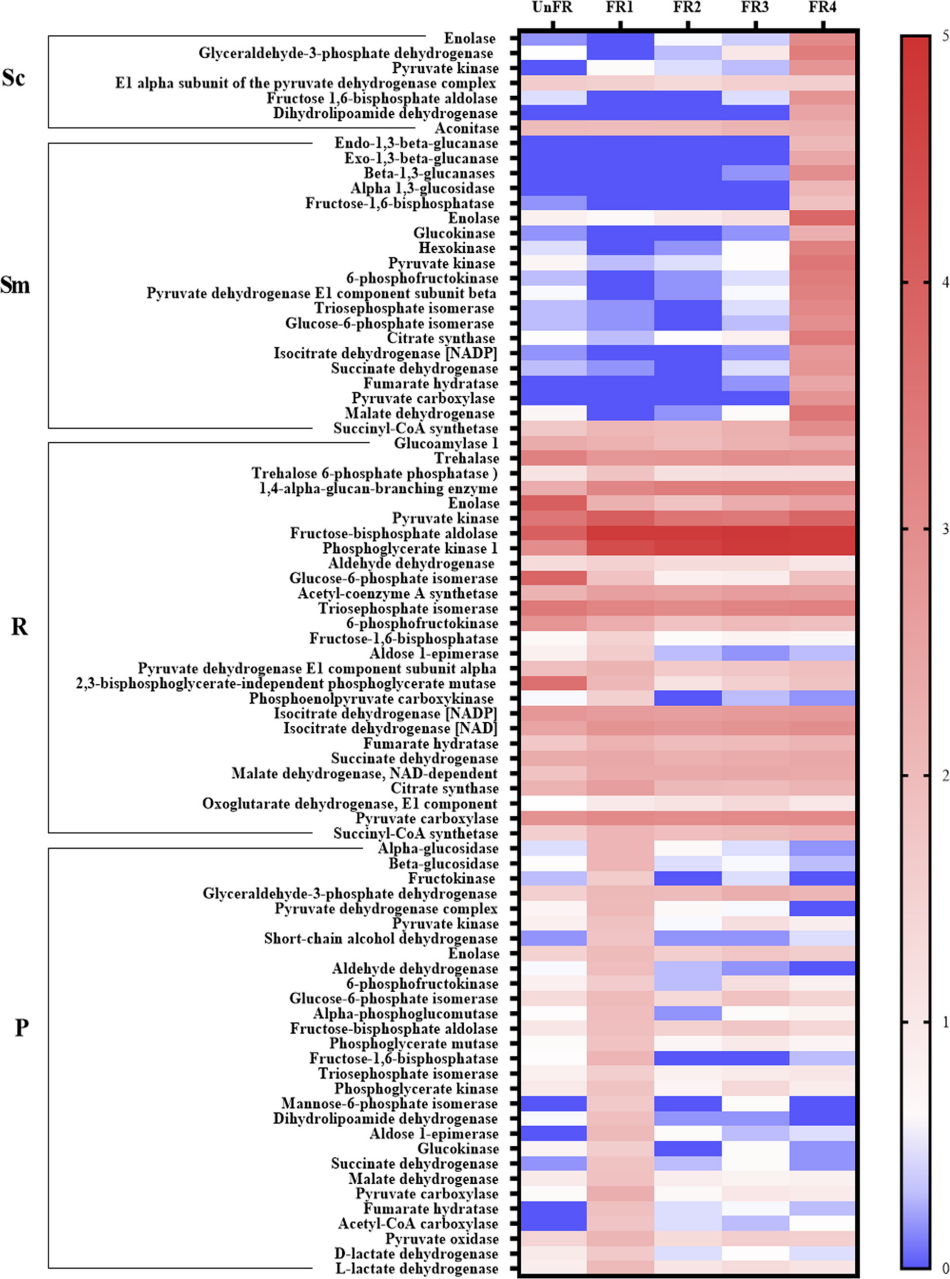
Relative expression level of enzymes related to carbohydrate metabolism in the FUBR samples. Four abundant carbohydrate metabolisms were analyzed: starch and sucrose metabolism, glycolysis/gluconeogenesis, the citrate cycle, and pyruvate metabolism. The relative expression was transformed to log_10_ values using the formular: log_10_[TPM + 1]. Sc, Sm, R, and P represent S. cerevisiae, *S. fibuligera*, *R. oryzae*, and P. pentosaceus, respectively.

Genes involved in carbohydrate metabolic pathways were analyzed based on the KEGG database. Several key carbohydrate metabolisms, including starch and sucrose metabolism, glycolysis/gluconeogenesis, pyruvate metabolism, and the citrate cycle pathway, were selected for analysis. As shown in Fig. 3, enzymes related to these pathways were present. The results indicated that the majority of enzymes in these pathways were upregulated in the FR samples. Most of the enzymes from *R. oryzae* and P. pentosaceus were highly expressed in the FR1 sample, whereas those from S. cerevisiae and *S. fibuligera* were highly expressed in the FR4 sample. Among these microorganisms, most of the enzymes from *R. oryzae* showed the highest expression level and tended to be expressed throughout the fermentation period.

**FIG 4:**
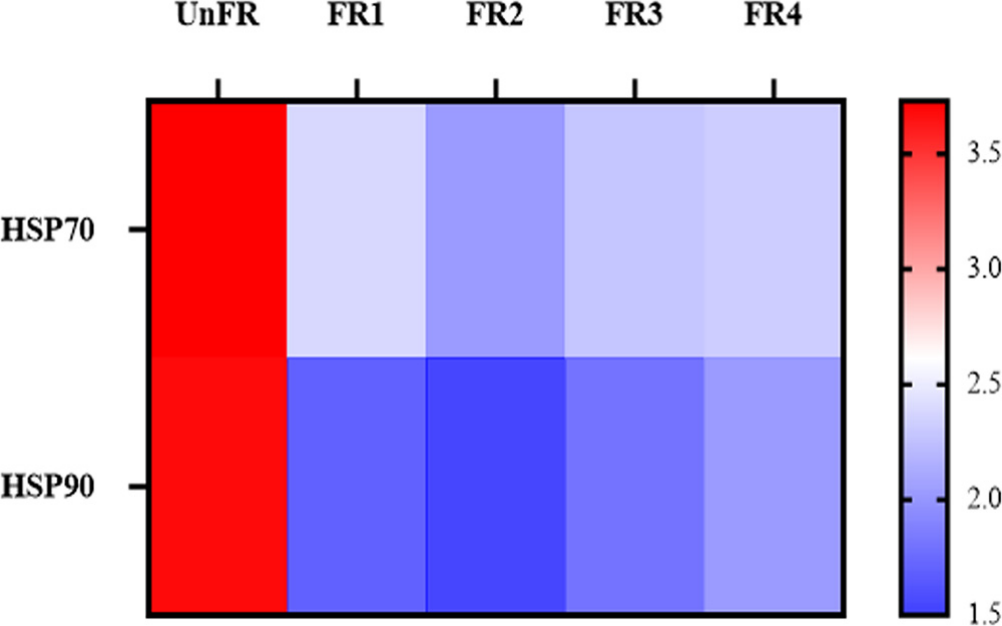
Relative expression level of heat shock protein (Hsp) 70 and 90 in the FUBR samples. The relative expression of HSP70 and HSP90 by *R. oryzae* was transformed to log_10_ values using the formular: log_10_[TPM + 1].

According to the KEGG annotation, for the starch and sucrose metabolism, mannosyl-oligosaccharide glucosidase, beta-glucosidase, exo-1,3-beta-glucanase, beta-1,3-glucanases, and alpha 1,3-glucosidase from *S. fibuligera*; 4-alpha-glucan-branching enzyme from *R. oryzae*; and alpha-glucosidase, beta-glucosidase, and fructokinase from P. pentosaceus were upregulated in the FR samples, although at different time points. Interestingly, *R. oryzae* expressed enzymes related to the level of trehalose (a melanogenesis inhibitor), such as trehalose 6-phosphate phosphatase and trehalase, whose expression levels were increased and decreased, respectively, in the FR samples (Fig. 3). These results were consistent with the expression level of heat shock protein 70 and 90, the negative regulator of the trehalose synthesis, in *R. oryzae* that were downregulated compared to those in the UnFR control (Fig. 4). Moreover, the metatranscriptomic results also revealed that the expression level of MS11, a transcription activator for the glucoamylase gene, in S. cerevisiae increased its expression level in the FR sample (data not shown).

Considering that pyruvate and acetyl-coA serve as important intermediates for biosynthesis of amino acids, fatty acids (FAs) and other metabolites, the FR samples showed a likely ability for converting glucose to the pivotal intermediates of pyruvate and acetyl-coA. Hexokinase, glucokinase, pyruvate kinase, and 6-phosphofructokinase, which represent key enzymes in the glycolysis pathway, all showed a high expression level in the FR samples, although 6-phosphofructokinase from *R. oryzae* showed a high expression level even in the UnFR, but slightly decreased after fermentation (Fig. 3). Acetyl-CoA, another key intermediate metabolite, produced by pyruvate dehydrogenase and dihydrolipoamide dehydrogenase were upregulated during fermentation. Moreover, genes involved in pyruvate metabolism, including lactate dehydrogenase, pyruvate oxidase, and acetyl-CoA carboxylase, originated from P. pentosaceus were highly expressed in the FR sample (Fig. 3).

As for the expression level of key enzymes related to the citrate cycle, including citrate synthase, aconitase, isocitrate dehydrogenase [NAD], succinyl-CoA synthase, succinate dehydrogenase, fumarate hydratase, and malate dehydrogenase, they all showed a high expression level in the FR samples (Fig. 3), and highly expressed members of these enzymes were mostly derived from *S. fibuligera* and *R. oryzae*.

### Identification of genes related to the production of amino acids or peptides.

As plenty of amino acids or peptides have several biological activities, including melanogenesis inhibition, we investigated genes involved in protein degradation and amino acid biosynthesis that could produce these substances in the FUBR.

**FIG 5:**
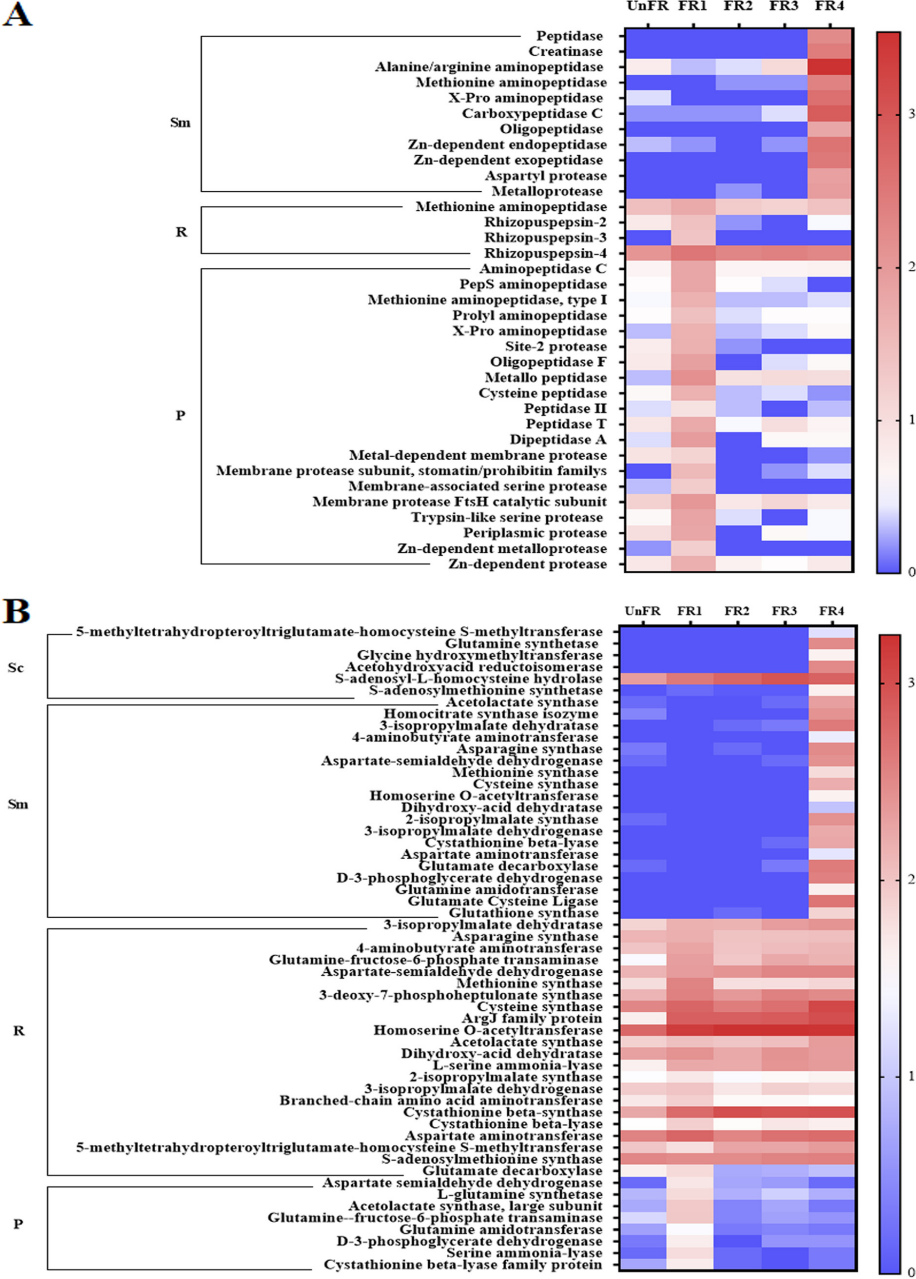
Relative expression level of enzymes related to peptides and amino acids biosynthesis in FUBR samples. Enzymes involved in (A) proteolysis and (B) biosynthesis of amino acids. The relative expression was transformed to log_10_ values using the formular: log_10_[TPM + 1]. Sc, Sm, R, and P represent S. cerevisiae, *S. fibuligera*, *R. oryzae*, and P. pentosaceus, respectively.

The metatranscriptomic results evidenced that several genes encoding proteolysis enzymes were found and mostly from P. pentosaceus followed by in *S. fibuligera* and *R. oryzae*, respectively. As shown in Fig. 5A, enzymes related to proteolysis were present. The transcription level of all these genes in P. pentosaceus, especially the metallo peptidase (MEROPS family M01) and membrane protease FtsH catalytic subunit, were abruptly increased in the FR1 sample and then rapidly decreased at later fermentation times. Similarly, the proteolytic enzymes in *R. oryzae*, especially rhizopepsin-4 and methionine aminopeptidase, were upregulated in the FR1 sample and then slightly decreased in later fermentation times, although they still showed high expression values. However, the expression level of the enzymes encoded by *S. fibuligera* were tremendously increased in the late fermentation stage (FR4) (Fig. 5A).

Further investigation was focused on the biosynthesis of amino acids with an emphasis on reactions involved in the metabolism of the defined microorganisms in the FUBR. In this study, we discovered diverse and abundant genes related to the biosynthesis of amino acids from the microorganisms in the FUBR, especially from *R. oryzae*. All the genes related to amino acid biosynthesis in these FR samples had a higher expression level than in the UnFR sample (Fig. 5B). Almost all of the genes obtained from *R. oryzae* and P. pentosaceus were upregulated in the FR1 sample, whereas genes obtained from S. cerevisiae and *S. fibuligera* were upregulated in FR4, except for S-adenosyl-L-homocysteine hydrolase that was upregulated since FR1 and remained upregulated in all the samples at all fermentation times (3 to 12 days). Among all the genes, those obtained from *R. oryzae* showed the highest expression level (Fig. 5B).

### Identification of genes related to the biosynthesis of fatty acids or unsaturated fatty acids.

Both fatty acids and unsaturated fatty acids have been reported to have a melanogenesis inhibition activity and be components in fermented rice (18). Therefore, we investigated the genes involved in lipolysis enzymes and the biosynthesis of unsaturated fatty acids.

**FIG 6:**
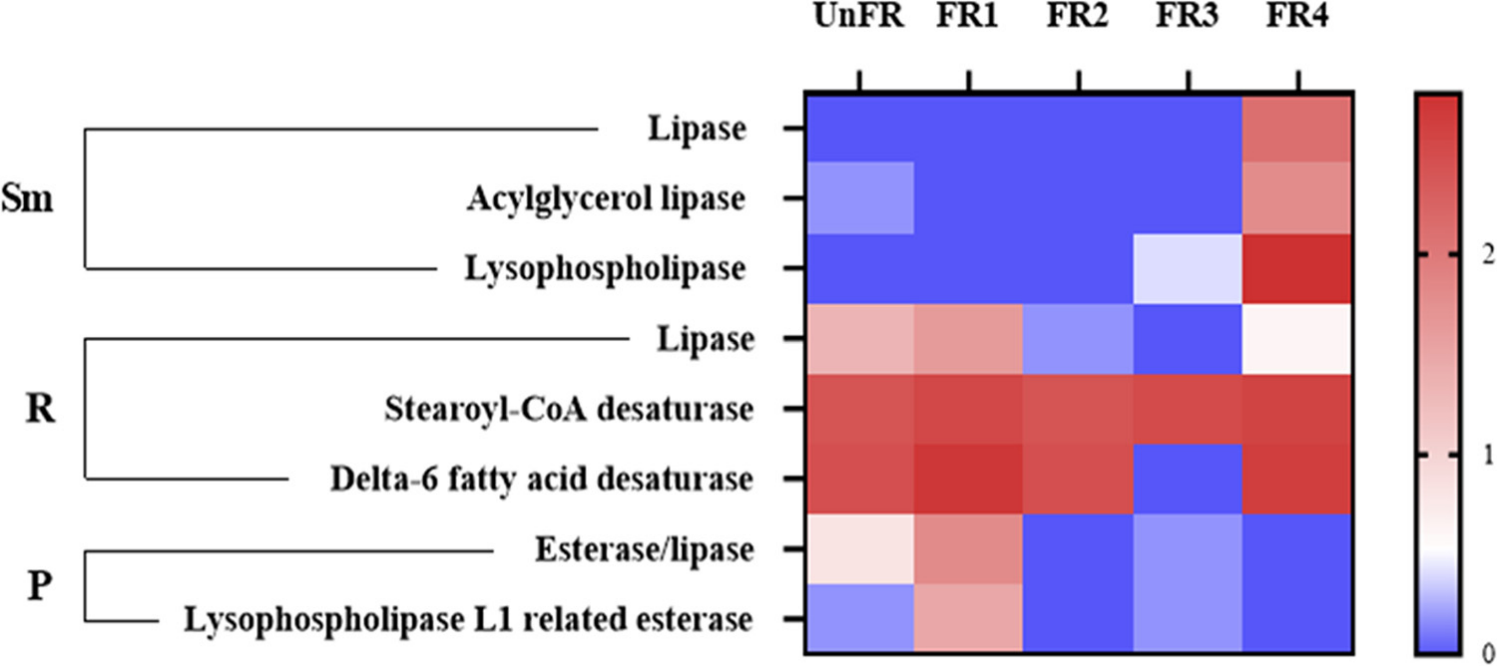
Relative expression level of enzymes related to fatty acid/unsaturated fatty acid biosynthesis in FUBR. Enzymes involved in lipolysis and biosynthesis of unsaturated fatty acid are shown as the relative expression level on a log_10_ scale (log_10_[TPM + 1]). Sm, R, and P represent *S. fibuligera*, *R. oryzae*, and P. pentosaceus, respectively.

Lipolysis enzymes catalyze the process of breaking down of lipids to fatty acids. The expression of lipolysis enzymes, such as lipase, acylglycerol lipase, and lysophospholipase, from *S. fibuligera*; lipase from *R. oryzae*; and esterase/lipase and lysophospholipase L1 related esterase from P. pentosaceus, were found to be upregulated in the FR samples (Fig. 6).

Besides lipolysis enzymes, the biosynthesis of unsaturated fatty acids, with an emphasis on reactions involved in microbial metabolism in the FUBR, was investigated. Acetyl-coA, an intermediate in carbohydrate metabolism, serves as an important intermediate for fatty acids biosynthesis leading to the biosynthesis of unsaturated fatty acids. The metatranscriptomic analysis revealed that biosynthesis of unsaturated fatty acids was indeed likely to have occurred. Only *R. oryzae* expressed the vital enzymes for unsaturated fatty acids biosynthesis, including stearoyl-CoA desaturase (SCD) and delta-6 fatty acid desaturase (FADS2), and both of these enzymes exhibited higher expression levels in the FR samples than in the UnFR sample (Fig. 6).

### Identification of genes related to carbohydrate transporters.

**FIG 7:**
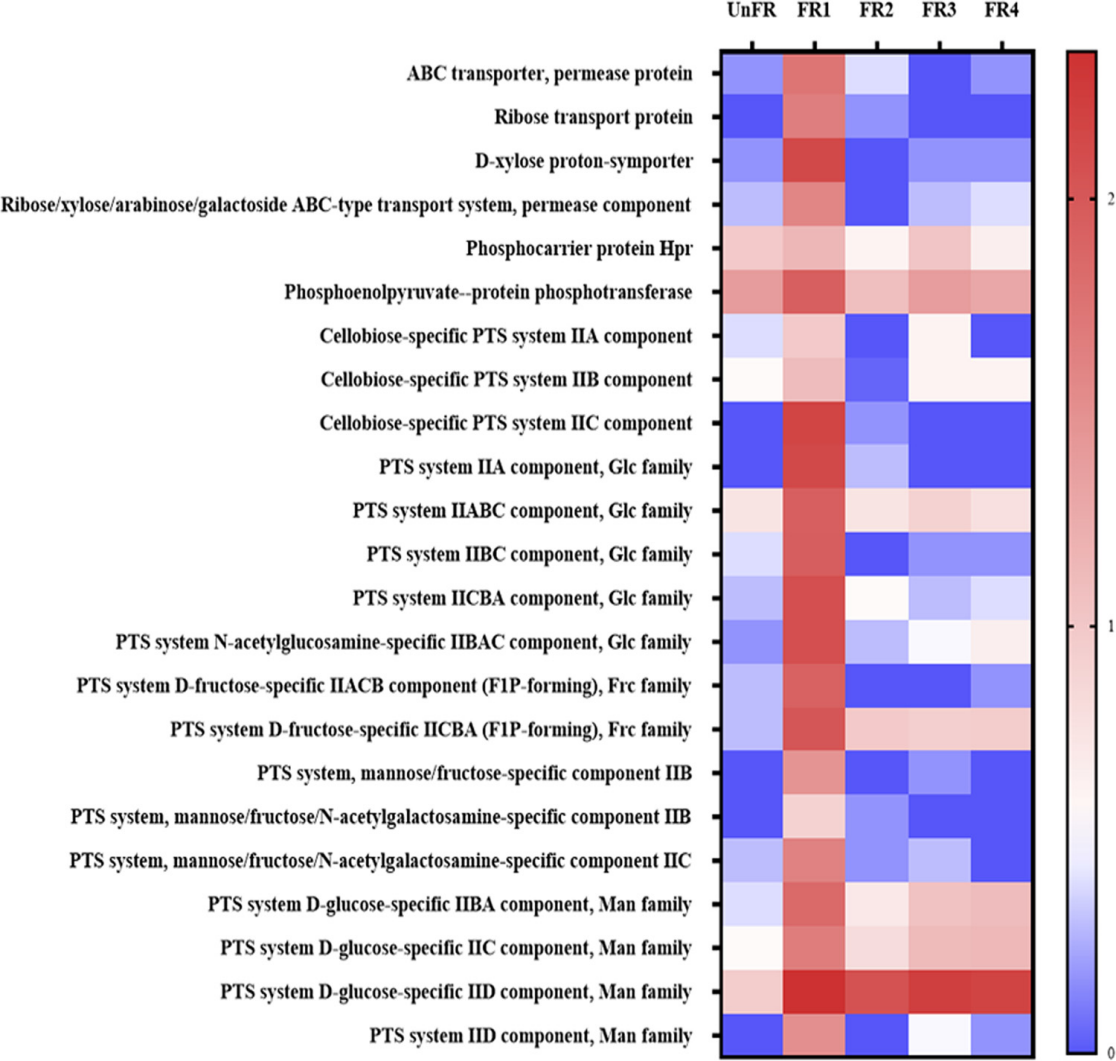
Relative expression level of enzymes related to carbohydrate transport in the FUBR samples. The relative expression was transformed to log_10_ values using the formular: log_10_[TPM + 1].

The highly efficient use of available carbohydrates is a prerequisite for microorganisms to flourish in a fermentation. Hence, the genes related to carbohydrate transporters were analyzed. The metatranscriptomic analysis showed that several genes related to carbohydrate transport systems were expressed by P. pentosaceus. Phosphotransferase transport system (PTS) was the major transport system in the FUBR, where the expression of genes encoding for the key PTS enzymes (phosphoenolpyruvate-protein phosphotransferase and phosphocarrier protein Hpr) and enzymes involved in various PTS transport families (glucose-glucoside [Glc], mannose-fructose-sorbose [Man], fructose-mannitol [Fru], and lactose-N, N’-diacetylchitobiose-β-glucoside [Lac]) were upregulated, especially in the early fermentation stage (Fig. 7). Among the FUBR samples, FR1 sample showed the highest expression level of all genes, especially those related to the PTS system d-glucose-specific IID component and the Man family, which showed the highest expression level in FR1 and then decreased in later fermentation times. However, their expression levels in the FR2–FR4 samples were still higher than in the UnFR (Fig. 7).

### qRT-PCR validation for the metatranscriptomic results.

**FIG 8:**
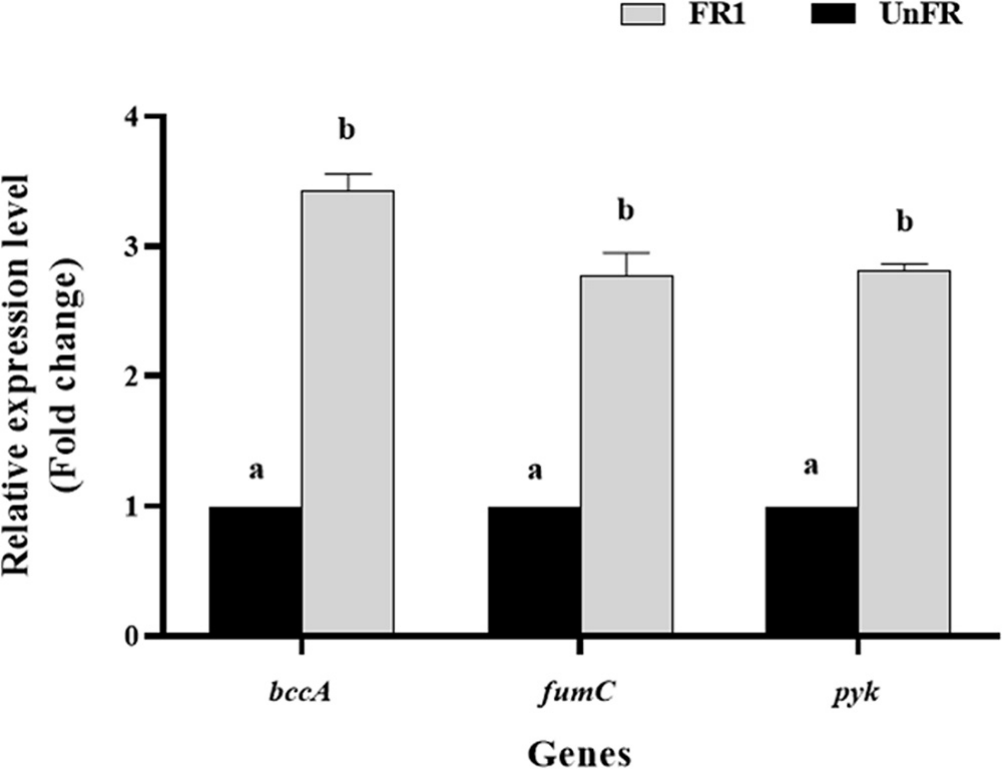
Relative expression levels obtained by qRT-PCR of acetyl-CoA carboxylase; *bccA*, fumarate hydratase; *fumC* and pyruvate kinase; *pyk*, of P. pentosaceus in UnFR and FR1 samples. The data are the mean ± SEM from three independent experiments. The bars marked with different letters are significantly different (*P* < 0.001) compared with the corresponding genes in the UnFR sample.

To confirm the changes in gene expression revealed by metatranscriptomic analysis, three different expressed genes of the UnFR and FR1 samples from P. pentosaceus (pyruvate kinase, fumarate hydratase, and acetyl-CoA carboxylase) were selected based on metatranscriptomic data for verification tests using qRT-PCR (Fig. 8). The transcript level of pyruvate kinase, fumarate hydratase and acetyl-CoA carboxylase in the FR1 sample were significantly increased (*P* < 0.001) compared with those in the UnFR sample, showing similar trend to the results of metatranscriptomic analysis.

### All four microbial species in the E11 starter are needed for the highest melanogenesis inhibition activity in the FUBR.

**FIG 9:**
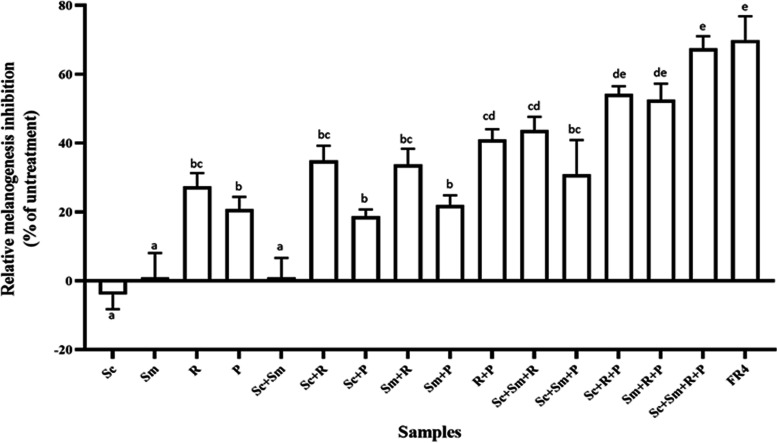
Melanogenesis inhibition activity of FUBR from different combinations of defined microorganisms in the E11 starter. B16F10 melanoma cells were treated with water (as control) or the indicated FUBR sample obtained from different combinations of the microorganisms isolated from E11 starter. Sc, Sm, R, and P represent S. cerevisiae, *S. fibuligera*, *R. oryzae*, and P. pentosaceus, respectively, compared to the FUBR from the E11 starter (FR4). Data are expressed as a percentage of the untreated control and presented as the mean ± SEM from three independent experiments performed in triplicate. Bars marked with different letters are significantly different (*P* < 0.05).

To determine the relationship between the core microorganisms in the E11 starter and the derived melanogenesis inhibition activity in the FUBR, the UBR was fermented with various combinations of the four microbial species from the E11 starter for 12 days and then screened for the melanogenesis inhibition activity against B16F10 cells. The metatranscriptomic results indicated that the *R. oryzae* and P. pentosaceus highly expressed transcripts for the enzymes involved in the production of melanogenesis inhibitors in the early stage (FR1) of FUBR production. In contrast, S. cerevisiae and *S. fibuligera* highly expressed these enzymes in the late fermentation stage. Therefore, to confirm these results, the UBR was fermented with various combinations of the four microbial species isolated from E11 starter for 12 days and then their melanogenesis inhibition activity against B16F10 cells was determined. The results revealed that *R. oryzae* (R) and/or P. pentosaceus (P), including the mixture of microbes containing these species, showed a different activity. However, the samples from the S. cerevisiae (Sc) and/or *S. fibuligera* (Sm) fermentations showed no activity. Among all of the samples, the FUBR from Sc + Sm + R + P showed the highest activity, and this was similar to the FUBR obtained from the E11 starter, with the others were ranked (highest activity to lowest) as Sc + *R + P* = Sm + *R + P* > Sc + Sm + R = *R + P* > Sc + R = Sm + R > Sc + Sm + *P* > R > *P* = Sm + *P* = Sc + P (Fig. 9). Although the activity of the FUBR obtained from the mixture of the four microbial species was not significantly different to that from Sc + R + P or Sm + R + P, it was numerically higher.

## DISCUSSION

In a fermentation process, microorganisms play critical roles in the characteristics of the obtained flavor and biological activities. A previous study found that the UBR fermented with E11 starter for 12 d showed a potent anti-melanogenesis activity (17), which was similar to that obtained in this study from the UBR fermented with mixture of the four species of microorganisms isolated from the E11 starter: S. cerevisiae, *S. fibuligera*, *R. oryzae*, and P. pentosaceus. Thus, the present study aimed to investigate the role of these four defined species in the E11 starter that produced the FUBR with a high melanogenesis inhibition activity by using metatranscriptomics to provide information on the metabolic processes and to help explain how the selected species in the FUBR influenced the production of compounds with melanogenesis inhibition activity.

The results showed that the FR samples had a strong melanogenesis inhibition activity against B16F10 cells in a fermentation-time-dependent manner, whereas the UnFR sample contained no such activity (Fig. 1). Thus, the melanogenesis inhibitor was derived from certain microorganisms in the fermentation of UBR.

This study focused on carbohydrate metabolism, biosynthesis of amino acids, proteolysis enzymes, lipolysis enzymes, biosynthesis of fatty acids/unsaturated fatty acids, and carbohydrate transporters, with an emphasis on reactions involved in the metabolism of substrates available in the fermented rice that could lead to the production of melanogenesis inhibitors, such as organic acids, amino acids, peptides, and fatty acids/unsaturated fatty acids (12, 19, 20).

**FIG 10:**
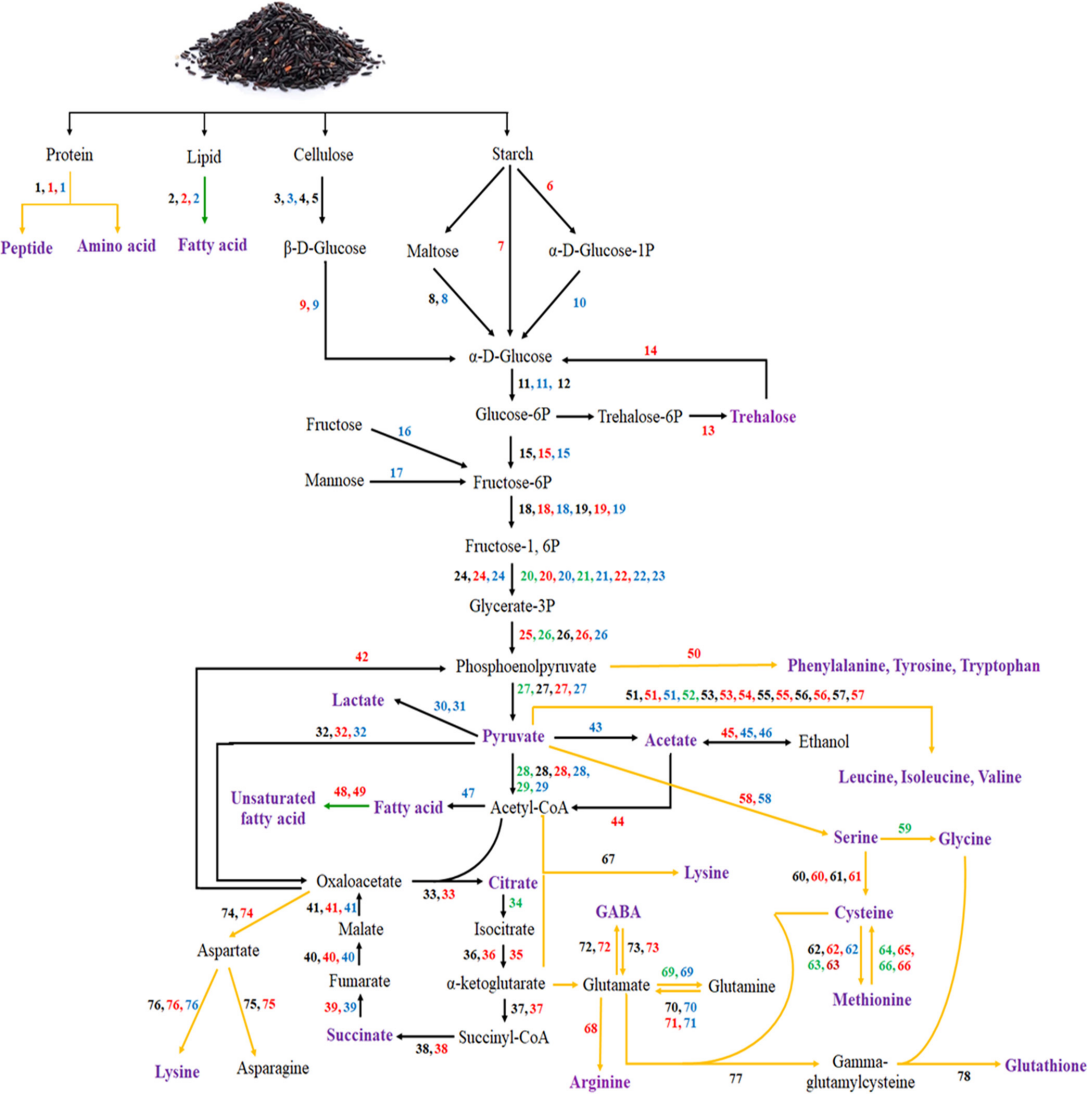
Schematic pathways inferred from the metatranscriptomics data of the UBR fermentation to produce melanogenesis inhibitors. The black, yellow, and green arrows indicate the pathway correlated with carbohydrate metabolism, biosynthesis of peptides/amino acids, and biosynthesis of fatty acid/unsaturated fatty acid, respectively. Purple letters represent compounds with melanogenesis inhibition activity. No.1 to 78 represent microbial enzymes of S. cerevisiae (green), *S. fibuligera* (black), *R. oryzae* (red), and P. pentosaceus (blue) in the FUBR detected the metatranscriptomic analysis. Proteolytic enzyme^1^, Lipolysis enzyme^2^, Beta-glucosidase^3^, Exo-1,3-beta-glucanase^4^, Endo-1,3-beta-glucanase^5^, 1,4-alpha-glucan-branching enzyme^6^, Glucoamylase 1^7^, Alpha-glucosidase^8^, Aldose 1-epimerase^9^, Alpha-phosphoglucomutase^10^, Glucokinase^11^, Hexokinase^12^, Trehalose 6-phosphate phosphatase^13^, Trehalase^14^, Glucose-6-phosphate isomerase^15^, Fructokinase^16^, Mannose-6-phosphate isomerase^17^, 6-phosphofructokinase^18^, Fructose-1,6-bisphosphatase^19^, Fructose-bisphosphate aldolase^20^, Glyceraldehyde-3-phosphate dehydrogenase^21^, Phosphoglycerate kinase^22^, Phosphoglycerate mutase^23^, Triosephosphate isomerase^24^, 2,3-bisphosphoglycerate-independent phosphoglycerate mutase^25^, Enolase^26^, Pyruvate kinase^27^, Pyruvate dehydrogenase E1 component subunit alpha^28^, Dihydrolipoamide dehydrogenase^29^, d-lactate dehydrogenase^30^, L-lactate dehydrogenase^31^, Pyruvate carboxylase^32^, Citrate synthase^33^, Aconitase^34^, Isocitrate dehydrogenase [NAD]^35^, Isocitrate dehydrogenase [NADP]^36^, Oxoglutarate dehydrogenase^37^, Succinyl-CoA synthetase^38^, Succinate dehydrogenase^39^, Fumarate hydratase^40^, Malate dehydrogenase^41^, Phosphoenolpyruvate carboxykinase^42^, Pyruvate oxidase^43^, Acetyl-coenzyme A synthetase^44^, Aldehyde dehydrogenase^45^, Short-chain alcohol dehydrogenase^46^, Acetyl-CoA carboxylase^47^, Stearoyl-CoA desaturase (SCD)^48^, Delta-6 fatty acid desaturase (FADS2)^49^, 3-deoxy-7-phosphoheptulonate synthase^50^, Acetolactate synthase^51^, Acetohydroxyacid reductoisomerase^52^, Dihydroxy-acid dehydratase^53^, Branched-chain amino acid aminotransferase^54^, 2-isopropylmalate synthase^55^, 3-isopropylmalate dehydratase^56^, 3-isopropylmalate dehydrogenase^57^, l-serine ammonia-lyase^58^, Glycine hydroxymethyltransferase^59^, Homoserine O-acetyltransferase^60^, Cysteine synthase^61^, Cystathionine beta-lyase^62^, 5-methyltetrahydropteroyltriglutamate-homocysteine S-methyltransferase^63^, S-adenosyl-L-homocysteine hydrolase^64^, Cystathionine beta-synthase^65^, S-adenosylmethionine synthase^66^, Homocitrate synthase isozyme^67^, ArgJ family protein^68^, l-glutamine synthetase^69^, Glutamine amidotransferase^70^, Glutamine–fructose-6-phosphate transaminase^71^, Glutamate decarboxylase^72^, 4-aminobutyrate aminotransferase^73^, Aspartate aminotransferase^74^, Asparagine synthase^75^, Aspartate semialdehyde dehydrogenase^76^, Glutamate Cysteine Ligase^77^, and Glutathione synthase^78^.

First, for genes involved in carbohydrate metabolism (starch and sucrose metabolism, glycolysis, pyruvate metabolism, and the citrate cycle) during rice fermentation. The sugar reduction produces organic acids (e.g., lactate, acetate, fumarate, succinate, malate, and pyruvate) including intermediates for the formation of other metabolites, such as amino acids and fatty acids or unsaturated fatty acids (Fig. 10) (9, 21–23) which have been reported to have a melanogenesis inhibition activity (18–20, 24). We discovered upregulated transcript levels in diverse and abundant genes related to carbohydrate metabolism enzymes in the FUBR samples, which is consistent with the concentration of organic acids in the ferment that are known to have melanogenesis inhibition activity (data not shown).

Trehalose is another metabolite from the fermentation process that has an antioxidant activity and can inhibit melanogenesis. It is normally used as an ingredient in cosmetics and skincare products (25–28). Previous reports showed that the HSP70 levels are a major controller of trehalose synthesis, while HSP90 and HSP104 play minor roles. The inactivation of Hsp70 genes led to the overproduction and slow degradation of trehalose (29, 30), which is consistent with our study where HSP70 and HSP90 from *R. oryzae* were highly downregulated during UBR fermentation (i.e., in the FR samples; Fig. 4). Likewise, this is consistent with the observation that genes involved in trehalose in *R. oryzae*, such as trehalose 6-phosphate phosphatase and trehalase, showed increased and decreased expression levels, respectively, (Fig. 3) leading to the accumulation of trehalose. After the substrate has been metabolized, the intermediates can be further used through glycolysis/gluconeogenesis, pyruvate metabolism, and the citrate cycle pathway.

The FR samples showed a significant potential for converting glucose to pivotal intermediates, such as pyruvate and acetyl-coA, which might then serve as direct or indirect substrates for other metabolites that have a melanogenesis inhibition activity. For example, lactate and acetate are two metabolites in fermented rice products that have a melanogenesis inhibition activity (12, 31, 32). Lactate dehydrogenase and pyruvate oxidase are key enzymes involved in the production of lactate and acetate, respectively, (Fig. 10) and those produced by P. pentosaceus were upregulated in the UBR fermentation process, especially at the early fermentation time (FR1; Fig. 3). The key enzymes related to the citrate cycle, including citrate synthase, aconitase, isocitrate dehydrogenase, succinyl-CoA synthase, succinate dehydrogenase, fumarase, and malate dehydrogenase (33), all showed high expression levels in the FUBR fermentation process leading to the production of other melanogenesis inhibitor, such as succinate and malate (data not shown).

Peptides or amino acids (such as alanine, glycine, isoleucine, and leucine), including the metabolites of amino acids (such as glutathione), have an excellent potential as antioxidants and have a melanogenesis inhibition activity (19, 34, 35). Several studies have reported that microbial fermentation is an effective method for the production of peptides or amino acids (7, 36–39). Hence, we analyzed the genes involved in the production of peptides or amino acids. Because protease (or peptidase) can be secreted by some microorganisms during fermentation, resulting in the accumulation of amino acids or peptides, we studied the expression level of genes involved in the degradation of protein in the fermented rice. The metatranscriptome analysis revealed that the upregulation of proteolytic enzymes was derived from (in order) P. pentosaceus, *S. fibuligera*, and *R. oryzae* (Fig. 5A), especially rhizopepsin-4 and methionine aminopeptidase from *R. oryzae*, which highly expressed throughout the fermentation period. Although the production of proteolytic enzymes occurred in the early fermentation period (day 3; FR1) and then slightly decreased after that (Fig. 5A), the highest proteolytic activity was reported in the acidic condition resulting from the fermentation process (40) and may lead to the increased peptide synthesis.

Besides proteolysis enzymes, we also analyzed enzymes involved in the amino acid biosynthesis pathway (Fig. 5B). The upregulated transcripts encoding for enzymes involved in the biosynthesis of amino acids was mostly derived from *R. oryzae*, with a minor participation of *S. fibuligera*, P. pentosaceus, and S. cerevisiae. This is consistent with a previous study that reported that fungi are equipped with powerful enzymes for both the degradation of protein into amino acids and for synthesizing amino acids (41, 42), resulting in an increased content of amino acids in the ferment during fermentation (7).

The expression of genes related to the synthesis of fatty acids/unsaturated fatty acids was also investigated, because they are known to have a melanogenesis inhibition activity (24, 43) and to increase in the rice fermentation (21). These include oleic acid (C18:1, n-9), stearic acid (C18:0), and linoleic acid (C18:2, n-6) that were synthesized after *Rhizopus* cultivation (44, 45). These are consistent with our metatranscriptomic analysis that indicated that *R. oryzae* showed highly expressed levels of the key enzymes (SCD and FADS2) involved in the synthesis of unsaturated fatty acids during the fermentation process. Moreover, lipolysis enzymes from *R. oryzae* and P. pentosaceus showed the highest expression level in the early fermentation stage (FR1), while those from *S. fibuligera* were highly expressed only in the late fermentation stage (FR4) (Fig. 6).

The utilization of carbohydrate by microorganisms requires their transport across the plasma membrane. Our metatranscriptomic analysis suggested that P. pentosaceus actively expressed genes involved in carbohydrate transport systems during the UBR fermentation process (Fig. 7). The PTS system, ABC transporter, permease, and symporter are known to be major carbohydrate transport systems in lactic acid bacteria (46), and was found to be the key carbohydrate transport system in this study, consistent with the previously reported results (47).

Furthermore, to confirm the changes in gene expression revealed by the metatranscriptomic analysis, P. pentosaceus genes (such as pyruvate kinase, fumarate hydratase, and acetyl-CoA carboxylase) from FR1 sample which had different expression levels from those in UnFR sample in metatranscriptomic data were selected as representative genes for verification by qRT-PCR. The results indicated that three selected genes from the FR1 sample experienced significant up-expression compared to those of the Un-FR sample, that was consistent with the metatranscriptomic data. Finally, to determine the relationship between the core microorganisms in the E11 starter and the formation of the melanogenesis inhibition activity in the FUBR, UBR was fermented with various combination of the four defined microbial species in E11 starter for 12 days and then screened for their activity. The FUBR obtained from *R. oryzae* and/or P. pentosaceus contained a certain activity level, whereas the FUBR from S. cerevisiae and/or *S. fibuligera* showed no activity unless *R. oryzae* and/or P. pentosaceus were also present (Fig. 9). These results are consistent with our metatranscriptomic results, which found that *R. oryzae* and P. pentosaceus were mostly active in the early fermentation stage (FR1) leading to the accumulation of melanogenesis inhibitors until day 12, whereas S. cerevisiae and *S. fibuligera* were active in the late fermentation stage. Therefore, all four of the microorganisms were required to obtain the FUBR with the highest melanogenesis inhibition activity.

Overall, our results indicated that the microorganisms in the E11 starter sequentially and/or coordinately synthesized the melanogenesis inhibitors leading to the accumulation of melanogenesis inhibitors in the FUBR. The function of the four microbial species in the E11 starter was inferred from the majority of enzymes from *R. oryzae* and P. pentosaceus exhibiting higher expression levels in the early fermentation stage (i.e., day 3) to produce diverse metabolites. Next, the condition or metabolites formed in the early stage induced the yeasts (S. cerevisiae and *S. fibuligera*) to express many related enzymes in the late fermentation stage (i.e., day 12).

The meta-pathways of the UBR fermentation by E11 starter were reconstructed based on the KEGG database and the metatranscriptomic data (Fig. 10). This schematic pathway was specifically related to carbohydrate metabolism (starch and sucrose metabolism, glycolysis/gluconeogenesis, pyruvate metabolism, and citrate cycle), amino acid biosynthesis, and fatty acid or unsaturated fatty acid biosynthesis. First, the biopolymers from UBR, such as cellulose, starch, protein, and lipid, are converted to monomers by diverse carbohydrate-active enzymes, proteolytic enzymes, and lipolysis enzymes. Next, the monomers are taken up and further utilized by the microbial community in the E11 starter. The products and intermediates of primary metabolism, such as glycolysis and the citrate cycle, as well as by-products of metabolism, contribute to the formation of compounds with a melanogenesis inhibition activity, such as trehalose, lactate, acetate, fumarate, succinate, malate, and pyruvate etc. In addition, the intermediates from the primary metabolism were also used to produce fatty acids or unsaturated fatty acids (such as oleic acid, stearic acid, and linoleic acid, etc.) and amino acids (such as alanine, glycine, isoleucine, and leucine etc.), including other metabolites (such as glutathione) that have melanogenesis inhibition activity.

### Conclusion.

In the present study, the UBR was fermented with the E11 starter to produce FUBR and determined for its melanogenesis inhibition activity. Moreover, the active microbial communities and their functional transcripts in the FUBR were evaluated using metatranscriptomic analysis. The results demonstrated that the melanogenesis inhibition activity of the FUBR was strongly affected by the fermentation time. Most of the upregulated gene transcripts from *R. oryzae* and P. pentosaceus were highly expressed in the early fermentation stage (day 3), while in the late fermentation stage, genes from S. cerevisiae and *S. fibuligera* might be induced from the early stage conditions to express enzymes that include those that form compounds with a melanogenesis inhibition activity. The qRT-PCR results from three representative genes of P. pentosaceus revealed consistent results with those in the metatranscriptomic data, thus verifying gene expression levels in our metatranscriptomic results. Furthermore, our metatranscriptomic results were also consistent with the melanogenesis inhibition activity in the FUBR obtained from fermenting with different combinations of the four principal species in the E11 starter, where these four microbial species likely sequentially synthesized compounds with a melanogenesis inhibition activity in the FUBR. This is potentially useful information for the quality improvement and technical optimization in producing the FUBR with the highest melanogenesis inhibition activity.

## MATERIALS AND METHODS

### UBR fermentation and sampling.

The UBR fermentation process was performed as previously described (17). Briefly, the UBR (purchased from Green Niche Rice, Thailand) was mixed with distilled water at a 1:2 (wt/vol) ratio and autoclaved at 121°C for 15 min. The cooked UBR was mixed with the E11 starter at 2% (wt/wt) and divided into five bottles, the lid was attached, and incubated at 30°C. The UnFR (unfermented) sample was harvested at the beginning of the FUBR production (day 0), while the FUBR product was harvested after 3, 6, 9, and 12 days of fermentation (called FR1, FR2, FR3, and FR4, respectively). Samples from each time point were stored at −80°C for subsequent metatranscriptomic analysis.

To determine its biological activity, the FUBR was centrifuged at 11,000 × *g* for 15 min to obtain the liquid supernatant, which was then used to screen for melanogenesis inhibition activity against the B16F10 melanoma cell line (17).

To determine which microorganisms in the E11 starter play a role in the production of the melanogenesis inhibition activity in the FUBR, the cooked UBR was fermented with each of the following combinations of the defined microorganisms isolated from the original E11 starter (S. cerevisiae (Sc), *S. fibuligera* (Sm), *R. oryzae* (R), *and* P. pentosaceus (P)), as per a previous study (17): (R + Sc, R + Sm, R + P, R + Sc + Sm, R + Sc + P, R + Sc + *P* + M, Sc + Sm, Sc + Sm + P), respectively. The fermented bottles were incubated at 30°C for 12 d, then the fermented liquid parts were harvested by centrifugation and determined for their melanogenesis inhibition activity against the B16F10 cell line.

### Ascertaining the melanogenesis inhibition activity of each FUBR sample.

Melanogenesis inhibition activity of each type of FUBR sample was determined by measurement of the melanin content in the B16F10 melanoma cell line as previously described (17). The B16F10 cell line (ATCCCCL-6475) was cultured in complete medium (CM; DMEM supplemented with 10% [vol/vol] fetal bovine serum, penicillin [100 U/mL], and streptomycin [100 mg/mL]) in six-well plates (5× 10^4^ cells/well) and incubated for 24 h at 37°C under a humidified 95% (vol/vol) air, 5% (vol/vol) CO_2_ atmosphere. The cells were then treated with 5% (vol/vol) of the liquid from the respective FUBR and incubated in CM as above for 72 h. After incubation, the cells were harvested and solubilized in 1 N NaOH at 60°C for 60 min, and the absorbance of the cell suspension was then measured by spectrophotometer at 405 nm (OD_405_). The melanogenesis inhibition was expressed as the relative melanogenesis inhibition (%) from equation:

Relative melanogenesis inhibition (%) = [1−(A÷B)/(C÷D)]× 100

where A and C are the OD_405_ values of the treated cells and untreated cells, respectively, and B and D are the protein concentrations of the treated cells and untreated cells, respectively.

### RNA extraction.

Total RNA was extracted from FUBR as previously reported (48) with some modifications. Briefly, 1 g of FUBR was homogenized into a fine powder in a precooled mortar with liquid nitrogen. Next, 4 mL of borate buffer at 80°C (200 mM sodium borate [pH 9.0], 30 mM ethyleneglycotetraacetic acid [EGTA], 1% [wt/vol] sodium dodecyl sulfate [SDS], 2% [wt/vol] polyvinylpyrrolidone [PVP], and 0.5% [vol/vol] Nonidet-40 (NP-40) combined in 0.1% [vol/vol] diethyl pyrocarbonate [DEPC]-treated water and then autoclaved; after cooling and just before use, 10 mM β-mercaptoethanol and 0.03% [vol/vol] RNase inhibitor were added) and 280 μL of proteinase K (20 mg/mL) were added. The mixture was incubated at 80°C for 2 min and centrifuged at 5,000 × *g* for 10 min. The supernatant was mixed with an equal volume of 70% (vol/vol) ethanol. The sample was applied to an RNeasy mini column and centrifuged for 5 min at 5,000 × *g*; this step was repeated for the residual sample. The sample was then cleaned following the RNeasy minikit protocol (Qiagen# 74104, Netherlands) and treated with DNase I (Fermentas, USA) according to the manufacturer's protocol. The concentration and purity of extracted RNA was determined using a NanoDrop2000 spectrophotometer (Thermo Fisher Scientific, USA). The RNA samples with an OD_260_/OD_280_ ratio greater than 1.8 were selected for deep sequencing.

### RNA-Seq library construction and sequencing.

The concentration of the total RNAs was measured using a DeNovix fluorometer (DeNovix). Sample purity was checked as above, while the integrity of the total RNAs was assessed using an Agilent 2100 Bioanalyzer (Agilent). Approximately 5 μg of total RNA from the respective sample was used to create individually indexed strand-specific RNA-Seq libraries using the QIAseq FastSelect RNA removal and QIAseq stranded total RNA library preparation kits (Qiagen). Eukaryotic and prokaryotic rRNAs were removed by adding 1 μL of QIAseq FastSelect–rRNA Yeast and QIAseq FastSelect–5S/16S/23S-rRNA bacteria removal solution (Qiagen, USA), and the reactions were subjected to fragmentation and cDNA synthesis. AMPure XP beads (Beckman Coulter Genomic) were used to separate the cDNAs from the reaction mix. Indexing adapters were ligated to the cDNAs, and subsequently all cDNA libraries were inspected for quality using the Agilent 2100 Bioanalyzer (Agilent) and quantified with a DeNovix fluorometer (DeNovix). The indexed sequencing libraries were pooled in an equimolar quantity and subjected to cluster generation and paired end 2 × 150 nucleotide read sequencing on an Illumina Hiseq sequencer. The corresponding sequence files were deposited in the NCBI’s Sequence Read Archive (SRA) under accession number PRJNA870967.

### Bioinformatics.

Bioinformatics analyses was comprised of an initial quality check of the raw reads data files using FASTQC software. The adapter and poor-quality reads were removed using Fastp (49). Postsequencing rRNAs were removed from downstream analyses using SortMeRNA (50). The expression of the remaining transcripts was quantified in each sample using Salmon (51) with the standard options. The quasi-mapping approach utilized by Salmon requires the reference transcriptomes following S. cerevisiae R64-1-1, P. pentosaceus ATCC 25745, *R. delemar* RA 99–880 (genetic and morphology very closely with *R. oryzae* [52]), and *S. fibuligera* KPH12 to build reference index to determine the position and orientation information for where the sequencing reads best map prior to quantification. A combined matrix of salmon’s estimate of the number of reads mapping to each transcript was then created to represent the transcript expression levels. TPM (transcript per million) was used for normalization, dividing the reads counts by the effective length of the transcript and per million scaling factor, respectively. The transcripts were functionally annotated by the KEGG database (53). Salmon outputs abundance estimates which predict the relative abundance of different isoforms. The relative expression was transformed to log_10_ values using the formular: log_10_[TPM + 1] (54) and visualized by heatmap using GraphPad Prism version 9 (GraphPad Software Inc., San Diego, CA, USA).

### Gene expression evaluation by reverse-transcription quantitative real-time PCR.

Total RNA from UnFR and FR samples were converted to cDNA and subjected for reverse-transcription quantitative real-time PCR (RT-qPCR) analysis. RT-qPCR was performed on CFX Connect real-time PCR system (Bio-Rad, CA, USA) using SsoAdvanced Universal SYBR green Supermix (Bio-Rad, CA, USA) in triplicates. The reaction condition was performed at 95°C for 3 min, followed by 40 cycles of 95°C for 30 s, primer specific temperature (49 to 54°C) for 30 s (Table 1) and 72°C for 30 s. The specific primers from P. pentosaceus used for determination of transcript levels are shown in Table 1. The mRNA levels of selected genes were normalized to that of 16s rRNA and expressed as relative abundance of mRNA transcripts using the formula 2^−ΔΔCT^.

**TABLE 1:** Oligonucleotide primers for reverse-transcription quantitative realtime PCR (RT-qPCR)

Gene	Primer sequence (5′–3′)	Annealing temp
Pyruvate kinase	AAAATCGTTTCAACACTTGGTC (Forward) ACTTGCCAGTAATCTTTTCTGC (Reverse)	51°C
Fumarate hydratase	ATCGCTGGATTAGAAGTTAATGCG (Forward) TTTTAGGGCAGCTTCTTTCAGC (Reverse)	53°C
Acetyl-CoA carboxylase	AGGCTTCGTCAAAGGTTGG (Forward) ATGTCGGCAGAATCCAGAC (Reverse)	54°C
16s rRNA	ACTTCCGTTAATTGATTATGACG (Forward) CATCCAGAAGTGATAGCAGAGC (Reverse)	49°C

### Statistical analysis.

The data are expressed as mean ± one standard error of the mean (SEM) and were analyzed using one-way ANOVA followed by Tukey’s method using the GraphPad Prism version 9 (GraphPad Software Inc., San Diego, CA, USA). A statistical probability of *P* < 0.05 was considered significant.
